# A Multiplex High-Resolution Melting (HRM) assay to differentiate *Fusarium graminearum* chemotypes

**DOI:** 10.1038/s41598-024-81131-5

**Published:** 2024-12-30

**Authors:** Lovepreet Singh, Milton T. Drott, Hye-Seon Kim, Robert H. Proctor, Susan P. McCormick, J. Mitch Elmore

**Affiliations:** 1 Department of Agronomy and Plant Genetics, University of Minnesota, St. Paul, MN 55108 USA; 2 Cereal Disease Laboratory, Agricultural Research Service, US Department of Agriculture, St. Paul, MN 55108 USA; 3 Department of Plant Pathology, University of Minnesota, St. Paul, MN 55108 USA; 4 Mycotoxin Prevention and Applied Microbiology, National Center for Agricultural Utilization Research, Agricultural Research Service, US Department of Agriculture, Peoria, IL 61604 USA

**Keywords:** Fusarium head blight, *Fusarium Graminearum*, Mycotoxins, Chemotype, High-resolution melting, Diagnostics, Fungi, Fungal genetics, Applied microbiology, Microbiology techniques

## Abstract

*Fusarium graminearum* is a primary cause of Fusarium head blight (FHB) on wheat and barley. The fungus produces trichothecene mycotoxins that render grain unsuitable for food, feed, or malt. Isolates of *F. graminearum* can differ in trichothecene production phenotypes (chemotypes), with individuals producing predominantly one of four toxins: 3-acetyldeoxynivalenol, 15-acetyldeoxynivalenol, nivalenol, or NX-2. Molecular tools to diagnose chemotypes remain inefficient. This study aimed to develop a single-tube, multiplex molecular assay that can predict the four *F. graminearum* chemotypes. Conserved functional regions of three trichothecene biosynthetic genes (*TRI1*,* TRI8*, and *TRI13*) were targeted to develop a high-resolution melting (HRM) assay. Multiplex HRM analysis produced unique melting profiles for each chemotype, and was validated on a panel of 80 isolates. We applied machine learning-based linear discriminant analysis (LDA) to automate the classification of chemotypes from the HRM data, achieving a prediction accuracy of over 99%. The assay is sensitive, with a limit of detection below 0.02 ng of fungal DNA. The HRM analysis also differentiated chemotypes from a small sample of *F. gerlachii*, *F. asiaticum*, and *F. vorosii* isolates. Together, our results demonstrate that this simple, rapid, and accurate assay can be applied to *F. graminearum* molecular diagnostics and population surveillance programs.

**Supplementary Information:**

The online version contains supplementary material available at 10.1038/s41598-024-81131-5.

## Introduction

Fusarium head blight (FHB) is a devastating disease of wheat, barley, and other cereal crops^1,2^. Although multiples species in the *Fusarium graminearum* species complex (FGSC) can cause FHB, *Fusarium graminearum* is the most important causal agent of FHB worldwide^3,4^. In addition to reduced yield, FHB-infected grain is typically contaminated with mycotoxins that can cause serious, adverse health effects when present in food or feed products^5–7^. FHB poses a consistent risk to global food security and food safety, causing multibillion dollar losses during epidemic years^8,9^.

**Fig. 1:**
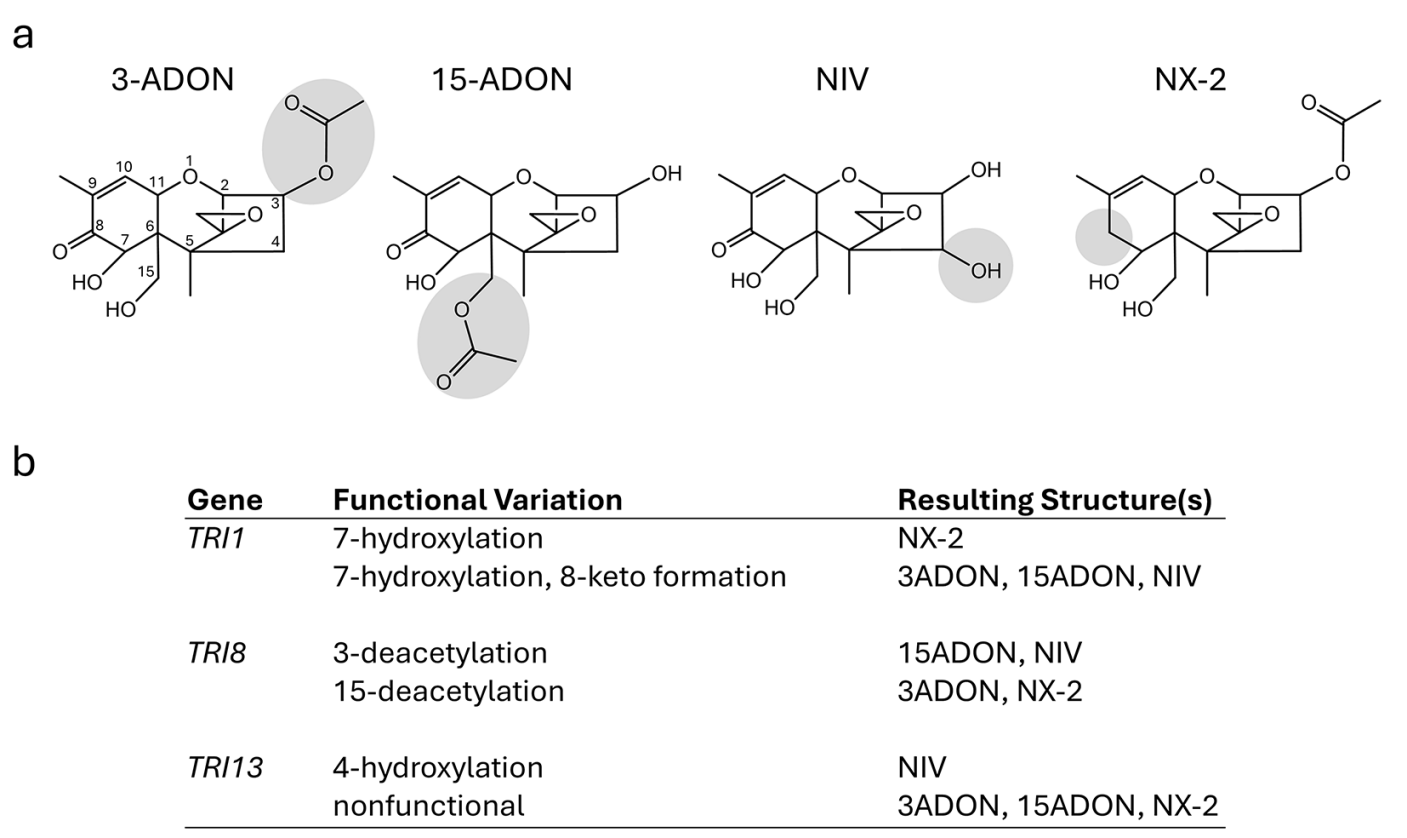
Genetic variation in trichothecene biosynthetic (*TRI)* genes determines structural differences in the trichothecene analogs produced by individual strains of *Fusarium graminearum*. (**a**) Key structural differences of predominant trichothecene analogs produced by *F. graminearum*: 3-acetyldeoxynivalenol (3-ADON), 15-acetyldeoxynivalenol (15-ADON), nivalenol (NIV), and NX-2. Numbers included in 3-ADON structure indicate positions in the trichothecene molecule. This numbering system is the same for all trichothecene analogs. The shaded regions on each structure highlight key structural differences. (**b**) Functional variation of the trichothecene biosynthetic genes *TRI1*, *TRI8* and *TRI13* that lead to key structural differences of *F. graminearum* trichothecene analogs.

Trichothecenes are the most notorious mycotoxins produced by *F. graminearum* (Fig. 1a). These sesquiterpenoid compounds act as virulence factors that enable FHB disease progression *in planta*^10–12^, and can cause a range of gastrointestinal, reproductive, and immune system disorders in animals^4,7^. Type B trichothecenes are characterized by the presence of a keto group at carbon atom 8 (C-8) (Fig. 1a). Historically, isolates of *F. graminearum* were thought to produce primarily one of three analogs of type B trichothecenes: either 3-acetyldeoxynivalenol (3-ADON), 15-acetyldeoxynivalenol (15-ADON), or nivalenol (NIV)^13–15^. However, recent studies demonstrated that some *F. graminearum* isolates instead produce a novel type A trichothecene analog known as NX-2. Unlike the type B trichothecenes described above, NX-2 lacks a keto group at C-8 but is otherwise structurally identical to 3-ADON (Fig. 1a)^16,17^. Like type B trichothecenes, NX-2 and/or its deacetylated derivative, NX-3, act as a virulence factor *in planta*^10^, and NX-3 exhibits similar cytotoxicity in eukaryotic cells^17,18^.

Trichothecene production phenotypes can differ within and among *Fusarium* species^4^. Chemotype designations often refer to the predominant trichothecene analog produced by a strain in culture. For example, 3-ADON, 15-ADON, and NX-2 strains produce predominantly 3-ADON, 15-ADON, and NX-2 in culture, respectively. *In planta*, 3-ADON, 15-ADON, and NX-2 are deacetylated to deoxynivalenol (DON), DON, and NX-3, respectively. However, NIV strains produce predominantly 4,15-diacetylnivalenol in culture, which is deacetylated to NIV *in planta*. Hence, *F. graminearum* isolates are classified into four major chemotypes: 3-ADON, 15-ADON, NIV, and NX-2^10,19^.

An understanding of chemotypic diversity is critical for FHB disease management and toxin monitoring in grain supplies. Different trichothecene analogs are reported as more effective virulence factors on specific plant hosts^10,11,20^, although host-chemotype interactions are still not well understood. At least five genetically distinct populations of *F. graminearum* exist in North America (NA): the NA1, NA2, NA3, Southern Louisiana (LA), and Gulf Coast (GC) populations. These genetic groups differ in chemotype composition, with NA1, NA2, NA3, and LA isolates typically producing 15-ADON, 3-ADON, NX-2, and NIV, respectively^19,21–23^. Isolates that have been documented from the GC population generally produce either 3-ADON or NIV, with a smaller percentage that produce 15-ADON^22,23^. These *F. graminearum* populations can also vary in their aggressiveness. For example, NA2 isolates are generally more aggressive with faster growth and higher toxin accumulation in wheat compared to NA1 isolates^15,24,25^. While it is not clear that trichothecenes are driving this aggressiveness, the nesting of chemotype variants within populations makes chemotype a valuable marker to track population shifts and to understand disease dynamics. Changes in the geographic distribution of chemotypes are increasingly reported in the USA^13,15,22^ and abroad, a phenomenon that some attribute to a changing climate^26,27^. Given recent shifts in the geography of different FGSC species and chemotypes, intensified monitoring of trichothecenes is needed to better protect food safety and food security in the USA and abroad.

Chemical analysis of mycotoxin production via high-pressure liquid chromatography (HPLC) or gas chromatography (GC) - mass spectrometry (MS) is the gold standard in chemotyping, but can be prohibitively expensive or laborious for large-scale screening efforts. To address this limitation, various DNA-based molecular assays have been developed to identify chemotypes by genotyping sequence variation in trichothecene biosynthetic (*TRI*) genes^23,28–36^. Functional variation of alleles of the trichothecene biosynthetic genes *TRI1*, *TRI8* and *TRI13* lead to key structural differences of 3-ADON, 15-ADON, NIV and NX-2 (Fig. 1b)^37^. All three genes encode enzymes; *TRI1* and *TRI13* encode cytochrome P-450 monooxygenases (Tri1 and Tri13), and *TRI8* encodes an esterase/deacetylase (Tri8). Tri1 catalyzes formation of an 8-keto and 7-hydroxyl of trichothecene biosynthetic intermediates in strains with a 3-ADON, 15-ADON or NIV chemotype^17^. As a result, 3-ADON, 15-ADON and NIV all have an 8-keto and 7-hydroxyl. However, Tri1 in NX-2 strains lacks the activity required for 8-keto formation, and as a result, NX-2 lacks an 8-keto but has a 7-hydroxyl^17^. Tri8 catalyzes 15-deacetylation of biosynthetic intermediates that have acetyl groups at both the 3 and 15 positions in 3-ADON or NX-2 strains^37,38^. As a result, 3-ADON and NX-2 lack a 15-acetyl but have a 3-acetyl. In 15-ADON and NIV strains, Tri8 catalyzes trichothecene 3-deacetylation of biosynthetic intermediates. As a result, 15-ADON and 4,15-diANIV (a precursor of NIV) lack a 3-acetyl but have a 15-acetyl. Tri13 catalyzes trichothecene 4-hydroxylation^39–41^. Tri13 is functional in NIV strains, and as a result, NIV has a 4-hydroxyl. In contrast, Tri13 is not functional in 3-ADON, 15-ADON and NX-2 strains due to multiple mutations in the *TRI13* gene. As a result, 3-ADON, 15-ADON and NX-2 lack a 4-hydroxyl. Thus, the genetic variation in alleles of *TRI1*, *TRI8*, and *TRI13* that impacts trichothecene structural diversity offers useful targets for DNA-based diagnostic assays to predict chemotype. Qualitative and quantitative genotyping assays have been developed by targeting *TRI1*, *TRI13*, and additional *TRI* genes with varying diagnostic accuracy compared to chemical analyses^30,32,33^. To date, there is no single-tube genotyping assay that can detect all four chemotypes of *F. graminearum*.

High-resolution melting (HRM) analysis is a simple, rapid, and cost-effective technique for molecular genotyping^42,43^ that has been applied to diagnostics of human^44^, animal^45^ and plant pathogens^46–48^. This method involves PCR amplification in the presence of a saturating fluorescent double-stranded DNA-binding dye and subsequent denaturation of the amplicon across a fine-scale temperature gradient to generate a specific melting profile^49,50^. The HRM profile, measured by the reduction in fluorescence, depends upon the amplicon’s sequence composition, length, and reaction conditions^43,49,51^. Unlike multiplexed qPCR, HRM genotyping is based on melting temperature (T_m_) differences, avoiding the need for expensive fluorescent probes. Recently, this technique has also been leveraged to identify the NX-2 chemotype^52^, replacing a multi-step PCR-based technique. However, there is still a need for an assay that can efficiently resolve all trichothecene chemotypes. The objective of the current study was to develop a multiplex HRM assay capable of differentiating all four chemotypes of *F. graminearum* in a single, high-throughput, and cost-effective assay. The assay was validated on a panel of 80 isolates and a supervised classification approach was applied to predict the chemotype group of individual isolates using the HRM data. We demonstrate that this assay is sensitive, accurate, and can be a valuable tool for molecular surveillance of FHB pathogen populations.

## Results

### Multiplex HRM assay design

**Fig. 2:**
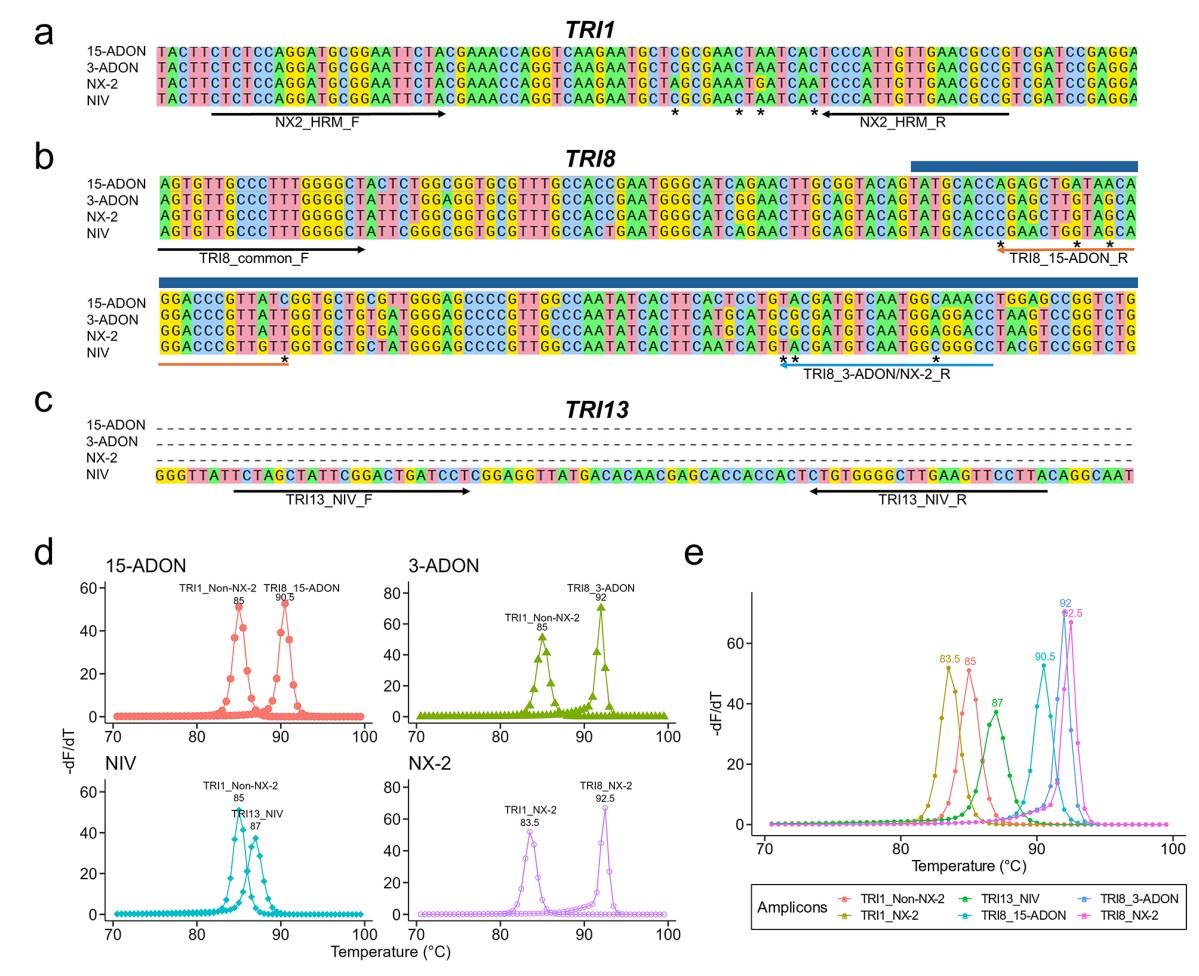
Design of a multiplex high-resolution melting (HRM) assay for the differentiation of the four trichothecene chemotypes of *Fusarium graminearum.* (**a**) A partial representative alignment of *TRI1* sequences from different chemotypes and the location of primers (arrows) used to generate NX-2 and non-NX-2 amplicons. (adapted from Singh et al.^52^). The SNPs targeted in the assay are marked with asterisks. (**b**) A partial representative alignment of *TRI8* sequences from different chemotypes and the location of primers (arrows) used to generate 15-ADON- and 3-ADON/NX-2-specific amplicons. The dark blue bar indicates part of a 306 bp functional region of *TRI8* that was previously identified as important for determining 15-ADON versus 3-ADON production^38^. The SNPs targeted to differentiate the binding of the reverse primers are marked with asterisks. (**c**) A partial representative alignment of *TRI13* where only NIV isolates have a ~ 178-bp insertion and the location of primers (arrows) used to generate a NIV-specific 75-bp amplicon. (**d**) Predicted derivative melt plots for each of the chemotypes in the multiplex HRM assay. Chemotypes are indicated in the top left corner of each plot. Single isolates are predicted to produce two amplicons. A single melting peak was predicted for each amplicon, therefore, there were two melting peaks per chemotype. Each peak is labeled with the source amplicon and predicted melting temperature. (**e**) Predicted derivative melt plots and melting temperatures of the six target amplicons in the multiplex HRM assay. The peaks are colored by the source amplicon. For (**d**) and (**e**), predictions were made using the uMELT Quartz software.

Three sets of oligonucleotide primers were used to differentiate the four *F. graminearum* chemotypes (Table 1). To distinguish NX-2 isolates from strains with other chemotypes, we targeted a region in the *TRI1* gene that contains four highly conserved NX-2-specific single nucleotide polymorphisms (SNPs), and the resulting amplicons distinguish NX-2 vs. non-NX-2 genotypes (3-ADON/15-ADON/NIV)^52^ (Fig. 2a). To differentiate 3-ADON isolates from isolates with the 15-ADON chemotype, primers were designed to target polymorphisms in a previously established functional region (649–954 bp) of the *TRI8* gene^38^. One common forward primer and two allele-specific reverse primers were designed to amplify either a 15-ADON-specific (102 bp) or 3-ADON/NX-2-specific (167 bp) *TRI8* region (Fig. 2b). To identify NIV isolates, we targeted a previously identified 178-bp insertion in the *TRI13* gene of NIV-producing isolates. This insertion is absent in the pseudogenized *TRI13* homologs of 15-ADON, 3-ADON, and NX-2-producing isolates^28,40,53^. Primers were designed to amplify a highly conserved 75-bp region within this insertion in NIV-producing strains and are not expected to produce an amplicon from isolates with the 3-ADON, 15-ADON or NX-2 chemotype (Fig. 2c).

**Table 1:** Primer sequences, target genes, and sizes of the resulting amplicons that were used in this study.

Target gene	Primer ID	Sequence (5’-3’)	Amplicon Size (bp)	Reference
*TRI1*	NX-2_HRM_F	CTCTCCAGGATGCGGAATTCTA		Singh et al. ^52^
	NX-2_HRM_R	CGGCGTTCAACAATGGGA	75	Singh et al. ^52^
*TRI8*	TRI8_common_F	AGTGTTGCCCTTTGGGGCT		current study
	TRI8_15ADON_R	GATAACGGGTCCTGTTATCAGCTCT	102	current study
	TRI8_3ADON/NX-2_R	GGTCCTCCATTGACATCGCG	167	current study
*TRI13*	TRI13_NIV_F	TCTAGCTATTCGGACTGATCCT		current study
	TRI13_NIV_R	TAAGGAACTTCAAGCCCCACAG	75	current study

Using the three primer sets in a single reaction, individual isolates were predicted to yield two amplicons with unique melting temperatures (T_m_) (Fig. 2d). Isolates with the 15-ADON chemotype should yield TRI1_non-NX-2 and TRI8_15-ADON amplicons, isolates with the 3-ADON chemotype should yield TRI1_non-NX-2 and TRI8_3-ADON amplicons, isolates with the NIV chemotype should yield TRI1_non-NX-2 and TRI13_NIV amplicons, and isolates with the NX-2 chemotype should yield TRI1_NX-2 and TRI8_NX-2 amplicons (Fig. 2d). The combination of the two amplicons for each isolate provides a unique HRM profile that should facilitate chemotype diagnosis. In total, there are six possible amplicons across the four chemotypes (Fig. 2e). The predicted derivative melt plots showed that each of the six amplicons had a single melting peak with a unique T_m_ (Fig. 2e). The amplicons TRI1_NX-2, TRI1_non-NX-2, TRI13_NIV, TRI8_15-ADON and TRI8_3-ADON, TRI8_NX-2 had predicted T_m_ of 83.5, 85, 87, 90.5, 92, and 92.5 °C, respectively (Fig. 2d-e). These predictions indicate that the HRM assay design was suitable for the multiplexed differentiation of all four chemotypes.

### Multiplex HRM assay performance on four reference isolates

**Fig. 3:**
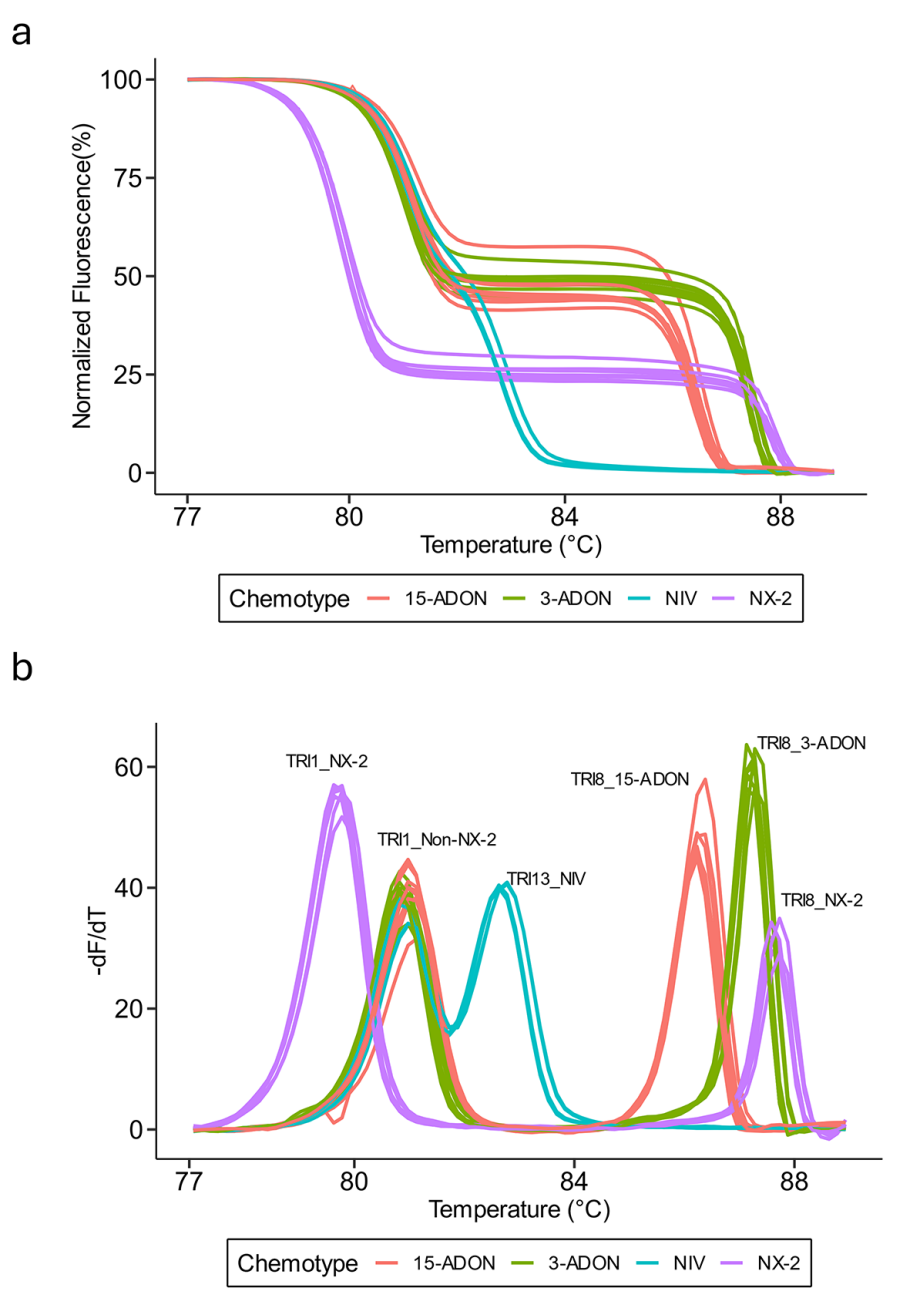
Multiplex HRM differentiates *Fusarium graminearum *chemotypes. (**a**) Normalized fluorescence plot derived from HRM analysis of *F. graminearum* isolates with each color representing a different reference isolate for each chemotype (PH-1, 15-ADON; 00-500, 3-ADON; 02–15, NIV; 06-156, NX-2). **(b)** Derivative melt plots of *F. graminearum* isolates representing each chemotype with the same coloration as **(a)**. Each melting peak is labeled with the identity of the corresponding amplicon. Eight technical replicates were included per chemotype during the multiplex HRM assay run.

The multiplex HRM assay was first tested using eight technical replicates on four reference isolates of *F. graminearum*, each representing one of the four chemotypes: 15-ADON (PH-1), 3-ADON (00-500), NIV (02–15), and NX-2 (06-156). The amplicons from each of the four chemotypes exhibited a unique normalized fluorescence HRM profile (Fig. 3a). As predicted by uMELT Quartz (Fig. 2d-e), each chemotype produced two amplicons and each amplicon generated a single melting peak with a unique T_m_ in the combined derivative melt plot (Fig. 3b). The melting peak patterns were similar to the in silico predictions by uMELT Quartz (Fig. 2d-e), although the experimental melting temperatures were shifted to lower values (Fig. 3b). The derivative melt plot of the 15-ADON chemotype exhibited the TRI8_15-ADON peak with a mean T_m_ of 86.27 °C and the TRI1_non-NX-2 peak with a mean T_m_ of 80.95 °C (Fig. 3b). The 3-ADON derivative melt plot had the TRI8_3-ADON peak with a mean T_m_ of 87.26 °C and the TRI1_non-NX-2 peak with a mean T_m_ of 80.95 °C (Fig. 3b). The NIV derivative melt plot contained the TRI13_NIV peak with a mean T_m_ of 82.69 °C and the TRI1_non-NX-2 peak with a mean T_m_ of 80.95 °C (Fig. 3b). The NX-2 derivative melt plot contained the TRI1_NX-2 peak with a mean T_m_ of 79.80 and the TRI8_NX-2 peak with a mean T_m_ of 87.67*°*C (Fig. 3b). The average T_m_ difference between the melting peaks across chemotypes ranged from 0.41 to 3.58 °C (Fig. 3b). Together, the multiplex HRM assay unambiguously differentiated each of the four chemotypes of *F. graminearum* (Fig. 3).

### Multiplex HRM assay validation

We validated the multiplex HRM assay on 80 FGSC isolates that were collected from diverse geographical regions and have been described previously (Supplementary Table S1). The panel included 23 15-ADON, 20 3-ADON, 16 NIV and 21 NX-2 isolates. The chemotypes of 48 isolates (*n* = 7, 15-ADON; *n* = 6, 3-ADON; *n* = 16, NIV; *n* = 19, NX-2) were previously determined by GC-MS, and the chemotypes of the remaining isolates were previously predicted by PCR-based genotyping (Supplementary Table S1). Chemotype assignments based on the HRM derivative melt plots matched the previously determined chemotypes for all *F. graminearum*,* F. gerlachii*, and *F. asiaticum* isolates (Supplementary Table S1). However, we noticed a shift in the 15-ADON-specific melting peak (TRI8_15-ADON) of the three *F. vorosii* isolates (NRRL 37605, 38207, 38208, all 15-ADON chemotype) (Supplementary Fig. S1). The HRM profile for these isolates still matched most closely to the reference 15-ADON profile (Supplementary Fig. S1).

**Fig. 4:**
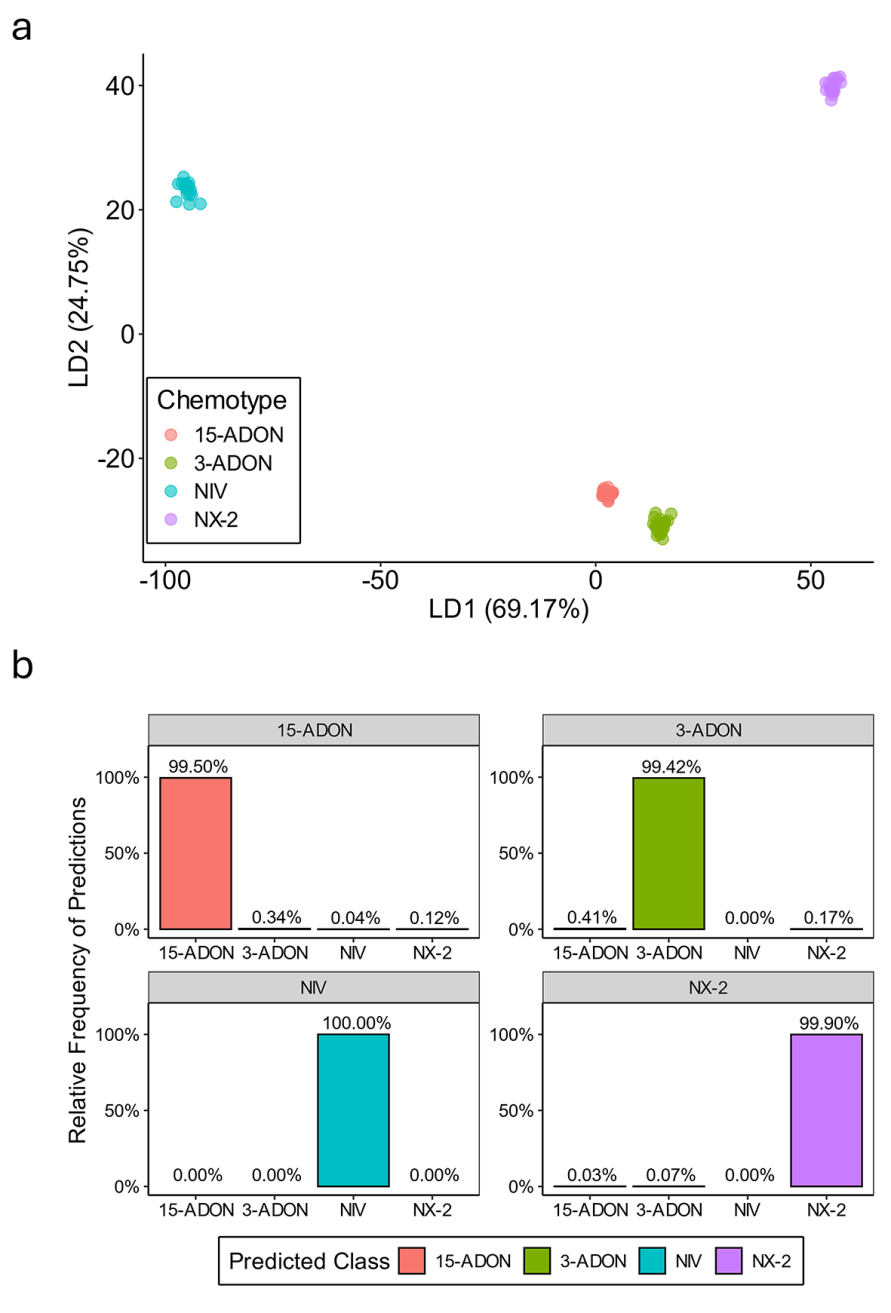
Validation of the multiplex HRM assay. (**a**) Linear discriminant analysis (LDA) plot of derivative melt data generated from the 80 isolates tested in this study. Each chemotype group is represented by a different color. The proportion of variance explained by the first (LD1) and second (LD2) linear discriminant functions is shown on the x- and y-axis, respectively. (**b**) Cross-validation results using 1,000 iterations of randomly selecting ~ 20% of the isolates to train an LDA classifier and using it to predict the chemotype class of the remaining isolates. Chemotype predictions are shown on the x-axis and the relative frequency of the predictions is portrayed on the y-axis. The gray bar at the top indicates the true chemotype assignment based on independent assays (Supplementary Table S1).

Next, we applied linear discriminant analysis (LDA) to the HRM data to obtain statistical confidence for the assignment of isolates to chemotype groups. LDA is a dimensionality reduction and supervised classification technique which has been employed for analyzing HRM data in previous studies^54–56^. Each chemotype class yielded a well-separated cluster in the LDA plot (Fig. 4a). The first and second linear discriminant functions (LD1 and LD2) explained 69.17% and 24.75% of the between-class variance in the dataset, respectively (Fig. 4a). Next, we used a cross-validation approach to assess the accuracy of chemotype classification using LDA and to estimate the robustness of chemotype discrimination using the HRM assay. We performed resampling for 1,000 iterations, in which each iteration a training set corresponding to ~ 20% of the isolates from each chemotype was randomly selected (*n* = 5, 15-ADON; *n* = 4, 3-ADON; *n* = 4, NIV; *n* = 5, NX-2). This stratified sampling strategy reflected the chemotype distribution of the larger dataset and ensured each chemotype was represented in the training set. In each iteration, an LDA classifier was built on the training data and used to predict the chemotype class of the test set comprised of the remaining ~ 80% of isolates. After 1,000 iterations, the overall prediction accuracy was 99.68%, with an average misclassification rate of 0.32%. The relative frequency of correct classification was 99.5% for 15-ADON, 99.42% for 3-ADON, 100% for NIV, and 99.9% for NX-2 (Fig. 4b). Collectively, these results suggest that the HRM assay is highly accurate and robust to deviations in the HRM profiles of individual isolates.

### Limit of detection of the assay

**Fig. 5:**
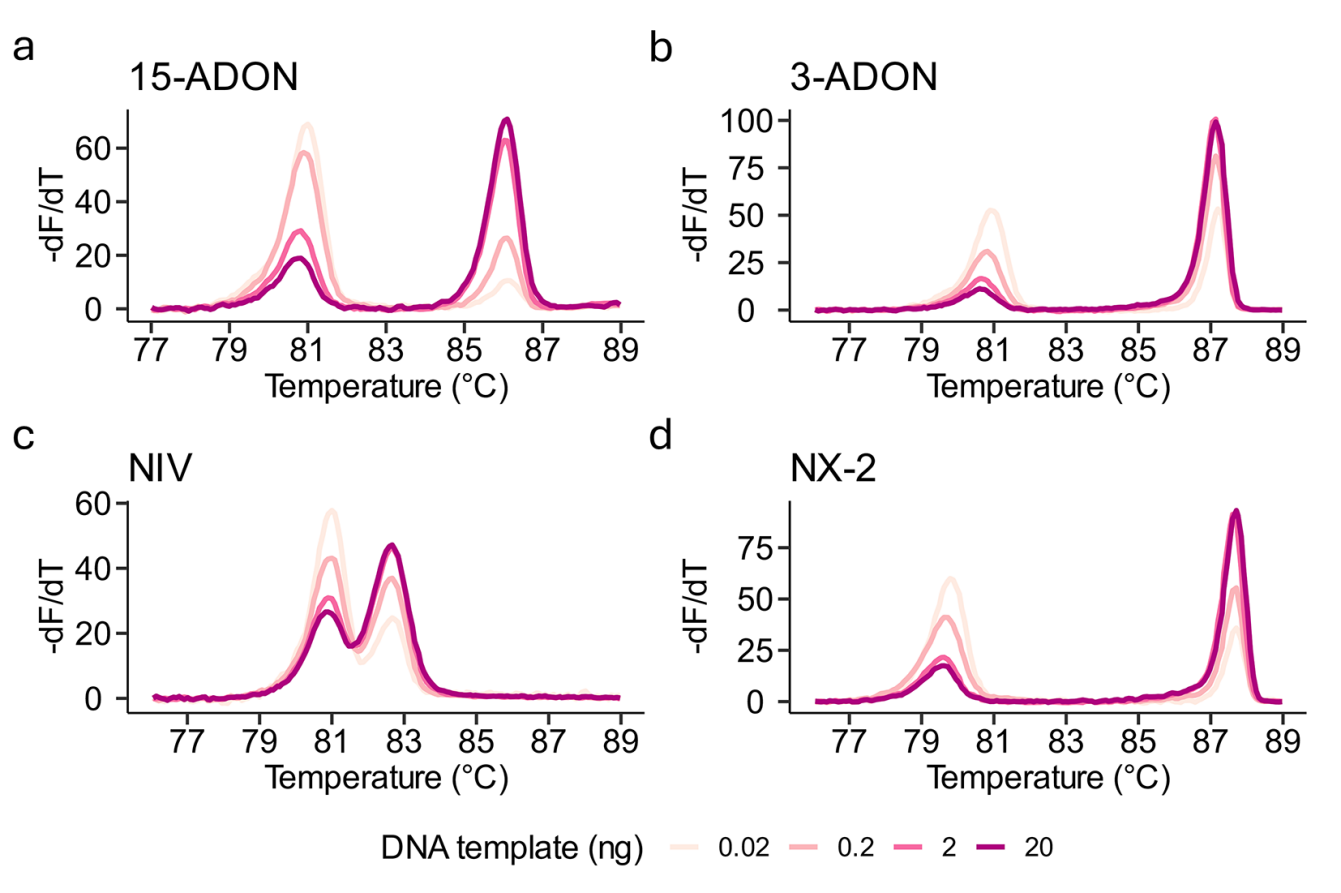
**Estimating the limit of detection (LOD) of the multiplex HRM assay.** Derivative melt plots of a ten-fold dilution series of template DNA (20, 2, 0.2, 0.02 ng) using DNA derived from **(a)** 15-ADON (strain PH-1), **(b)** 3-ADON (isolate 00-500), **(c)** NIV (isolate 02–15) or **(d)** NX-2 (isolate 06-156) reference isolates. A fifth concentration of 0.002 ng was used for all isolates but failed to amplify suggesting a LOD between 0.02 and 0.002 ng.

To demonstrate the sensitivity of the multiplex HRM assay, the limit of detection (LOD) was determined using a range of DNA templates from the four reference isolates in a 10-fold dilution series from 20 to 0.002 ng. For these sensitivity tests, we increased the PCR extension time to 15s (from 10s) and the number of cycles to 35 (from 30). Although the height of the melting peaks varied, the T_m_ for all melting peaks for 15-ADON, 3-ADON, NIV and NX-2 was consistent across a range of 20–0.02 ng DNA template (Fig. 5a-d). There was no amplification in reactions using 0.002 ng of DNA template, suggesting a LOD between 0.02 and 0.002 ng of pure *Fusarium* DNA.

## Discussion

FHB threatens small grain production in cereal-growing regions worldwide. Field surveys over the last 20 years have uncovered shifts in pathogen populations and dominant mycotoxin chemotypes in multiple areas^13,26^. Continuous monitoring is necessary to track population dynamics and to maintain an understanding of current mycotoxin threats to a given region. Genotyping assays have enabled large-scale surveys of chemotype variation in *F. graminearum* populations^15,23,30,57,58^. However, contemporary genotyping assays are either low-throughput, requiring multiple steps including PCR, restriction digestion, and gel electrophoresis, or require more costly reagents and equipment. To facilitate monitoring efforts and future epidemiological studies, we developed a single-tube, high-throughput, multiplex HRM assay that differentiates all four chemotypes of *F. graminearum*.

Targeting functional variation is more likely to result in a robust genotyping assay. In the present study, we targeted the genes responsible for production of structurally distinct analogs of trichothecene mycotoxins. Specifically, our assay targets conserved polymorphisms in *TRI8* to differentiate 3-ADON and 15-ADON^38^, *TRI13* to identify NIV^40^, and *TRI1* to identify NX-2^16,17,59^. Previously, genotyping assays have been developed by targeting sequence variation in *TRI1*^16,52,60,61^ and *TRI13*^28,34,62,63^. To our knowledge, this is the first report of a chemotype genotyping assay that targets the previously described functional region of *TRI8*^38^. Pasquali and Migheli suggested that genotyping based on *TRI8* for 3-ADON and 15-ADON chemotype prediction could be valuable given the inconsistent performance of assays developed from other *TRI* genes^30^.

In the present study, we validated the multiplex HRM assay for chemotype determination using 59 isolates of *F. graminearum*. In addition, we tested a small number of isolates of *F. asiaticum*, *F. gerlachii* and *F. vorosii*, all of which are members of the *F. graminearum* species complex (FGSC)^4^. The melting profiles of the *F. asiaticum* and *F. gerlachii* isolates matched closely with the reference isolates of *F. graminearum*, but deviations were observed with the three *F. vorosii* isolates. While the *F. vorosii* TRI8_15-ADON peak was shifted, the HRM profile still matched most closely to the other 15-ADON isolates. This observation suggests that our HRM assay may produce variant melting profiles in non-*F. graminearum* members of the FGSC due to additional sequence polymorphisms. Hence, further validation of the HRM assay is recommended for species other than *F. graminearum*. Nonetheless, our testing of a limited number of *F. asiaticum*, *F. gerlachii* and *F. vorosii* isolates suggests the potential utility of the HRM assay across FGSC species. These results also highlight an advantage of HRM genotyping, because it can be used for mutation discovery and variant scanning in PCR amplicons^64,65^. Such variants may not be identified using traditional PCR or qPCR assays. Thus, HRM can not only be used to identify known chemotypes but can also identify novel genotypes. Such genotypic variants may be particularly important when occurring in the functional regions of genes that impact trichothecene production.

Genotyping results of multiplex HRM assays are typically scored by visual data analysis. Automated genotype assignment of HRM data can provide statistical confidence, reduce bias, and further improve throughput^66^. A supervised machine learning-based dimensionality reduction and classification method, Linear Discriminant Analysis (LDA), has been used to automate HRM analysis on other systems^54,55^ including blue crab genotyping where it achieved > 90% accuracy differentiating between five species^56^. In our case, LDA analysis generated four distinct chemotype clusters and predicted the chemotypes with 99.68% accuracy. We speculate that, in contrast with many animal systems, LDA may be more accurate in fungi that exhibit lower diversity, particularly among clonal or partially clonal lineages. Regardless, the importance of visual data analysis cannot be negated. For instance, variation in the melting profile of *F. vorosii* isolates was only identified by visual inspection while LDA clustered these samples tightly with other 15-ADON isolates. Hence, the best approach to HRM scoring combines both automated and visual data analysis^66^.

Multiplex PCR assays, while cost-effective, tend to be less sensitive than single-plex PCR^67^. Previous multiplex HRM assays, such as the assay used to detect pathogens of strawberry crown rot^68^, have reported limits of detection (LOD) ranging from 0.1 ng to 0.001 ng of DNA template. In the present study, the LOD was estimated to be between 0.02 and 0.002 ng of pure *F. graminearum* DNA, which falls in the middle of the LOD range reported by others. In FHB field collections, a range of 0.3 ng – 199 ng of *F. graminearum* DNA per 100 mg of wheat grain tissue has been reported^69^. Given these estimates, we speculate that our multiplex HRM assay can be used for screening infected tissue samples without the labor-intensive isolation of pure fungal cultures. However, because inferences of chemotypic diversity are typically performed on pure cultures, where DNA concentrations are not a limiting factor, determining the utility of this assay *in planta* was beyond the scope of our efforts.

To our knowledge, this is the first report of a single, multiplex genotyping assay that can differentiate all four trichothecene chemotypes of *F. graminearum*. This rapid, accurate, and cost-effective assay is ideal for determining the chemotypes of a large number of isolates, such as those collected from field surveys. The importance of large-scale surveillance is increasingly important as a changing climate drives alterations in the distribution of plant pathogens^27,70,71^. Furthermore, the HRM method has the potential to uncover novel genetic variation that could impact trichothecene production phenotypes. Overall, this multiplex HRM assay should be valuable for monitoring *F. graminearum* population dynamics and can contribute to integrated disease and toxin management for FHB.

## Materials and methods

### Fungal cultures used in assay development

Fungal isolates used in this study were primarily *F. graminearum* (*n* = 59), with additional members of the FGSC: *F. asiaticum* (*n* = 5), *F. gerlachii* (*n* = 3), *F. vorosii* (*n* = 3), and 10 isolates without species determination (Supplementary Table S1). These isolates were previously collected from diverse geographic locations across the United States (*n* = 48), Canada (*n* = 39), Japan (*n* = 2) and Hungary (*n* = 1). Chemotypes were determined previously using GC-MS (*n* = 48) or PCR genotyping (*n* = 32) (Supplementary Table S1)^15,16,19,22,23,57^. Fungal strains were maintained as frozen glycerol stocks at the USDA Cereal Disease Lab, Saint Paul, MN, USA. A single *F. graminearum* isolate from each chemotype was used for initial assay development (PH-1 [NRRL 31084] for 15-ADON; 00-500 [NRRL 46416] for 3-ADON; 02–15 for NIV; 06-156 [NRRL 66038] for NX-2). The remaining 76 isolates were used for assay validation.

### Fungal DNA extraction and quantification

Frozen fungal cultures were revived on half-strength potato dextrose agar (PDA) at 25 °C with a 12/12-hour light and dark cycle for 7 days. To obtain tissue for DNA extractions, a 10 µl inoculation loop was used to transfer a small amount of mycelia to liquid complete medium^72^ and incubated at 25 °C with a 12/12-hour light and dark cycle while shaking at 150 RPM for 7 days. Mycelia were then harvested, washed with sterile water, lyophilized in 2 ml tubes, and stored at -80 °C until DNA extraction. Lyophilized fungal tissue was ground to fine powder by adding 0.5 mm silica beads and using a bead beater for 1 min. DNA was extracted from the homogenized fungal tissue using LETS buffer (100 mM lithium chloride, 20 mM EDTA, 10 mM Tris-HCL, pH 8.0 and 0.5% SDS) as described previously^73^ and resuspended in 10 mM Tris-HCl (pH 8.0). DNA samples were quantified using the Qubit dsDNA Broad Range Assay kit (Invitrogen, Carlsbad, CA, USA).

### HRM assay design

The *TRI1*, *TRI8*, and *TRI13* genes were targeted to develop the multiplex assay. For *TRI1*, we used the NX-2 HRM assay primers from our previous study^52^. To develop primers for *TRI8* and *TRI13*, we obtained whole-genome sequences from 328 genomes as part of a larger genome sequencing project^74^. Sequences of *TRI8* (NCBI accession AF359361) and *TRI13* (NCBI accession AY057841.1) genes were used as queries against the reference genomes using BLAST from the BLAST + suite v2.12.0 + ^75^. Two 100 bp windows, one on either side of the resulting hits were taken to help anchor genes with large deletions and the resulting regions were extracted from their corresponding genomes using bedtools ‘getfasta’ v.2.30.0^76^. Extracted gene sequences were aligned using the MAFFT algorithm in the SnapGene software version 7.0.2 (http://www.snapgene.com). Primers were designed in SnapGene to target the conserved and chemotype-specific polymorphisms. The primers used in the study are listed in Table 1. The uMELT Quartz software^77^ (version 3.6.2), a web-based application, was used for in silico prediction of melting profiles for the amplicons of all four chemotypes in the multiplex assay.

### High Resolution Melting assay

Multiple primer concentrations and thermal cycling parameters were tested to develop the assay, and below we report only the optimal conditions as run on DNA from all 80 isolates. Each 20 µl reaction contained 10 µl of 2× LightCycler 480 High Resolution Melting Master Mix (Roche Lifesciences, Indianapolis, IN, USA), 0.15 µl of 10 µM each NX2_HRM_F and NX2_HRM_R, 0.6 µl of 10 µM TRI8_common_F, 0.3 µl of 10 µM each TRI8_15ADON_R and TRI8_3ADON/NX-2_R, 0.2 µl of 10 µM each TRI13_NIV_F and TRI13_NIV_R, 1.2 µl of 25 mM MgCl_2_, and 2.5 µl of 10 ng/µl DNA template (Table 1). Molecular biology grade water was used as a non-template control (NTC). HRM assays were performed with the Roche LightCycler 480 II instrument (Roche Lifesciences, Indianapolis, IN, USA) using a single thermal cycling protocol where PCR was followed directly by HRM analysis. The PCR was performed by incubating samples at 95 °C for 10 min followed by 30 cycles of denaturation at 95 °C for 10 s, annealing at 60 °C for 15 s, and extension at 72 °C for 10 s. Subsequently, PCR reactions were incubated at 95 °C for 1 min followed by incubation at 40 °C for 1 min. HRM was then performed by increasing the temperature from 65 °C to 95 °C at a ramp rate of 1 °C per second with 25 fluorescence acquisitions per second. Initial assay development on the four reference isolates was performed with eight technical replicates. All 80 isolates were assayed in a single plate at once without technical replicates and the assay was repeated twice with similar results. The raw fluorescence data was exported from the LightCycler 480 II software and analyzed as described below.

### Data analysis

The raw fluorescence data were analyzed using the web-based software, uAnalyze 2.1^78^, and R statistical software (v4.1.0; R Core Team 2021). Using uAnalyze 2.1, the raw dataset was narrowed to a temperature range of 76.6 °C to 89 °C (Supplementary File S2. The raw relative fluorescence unit (RFU) values were normalized using the linear baseline extrapolation method of background subtraction in uAnalyze 2.1 (Supplementary File S3). Derivative melt plots displaying the rate of change in fluorescence relative to temperature were compared visually to the derivative melt plots of the reference isolates to determine the genotypes of the remaining isolates. The derivative melt data (Supplementary File S4) were imported into R for downstream data analyses. We predicted chemotype classes from the HRM data using linear discriminant analysis (LDA) from the R packages MASS (v7.3.58.3)^79^ and caret (v6.0-94)^80^. First, LDA was applied on the entire HRM dataset and the first (LD1) and second (LD2) linear discriminants for each isolate were plotted to evaluate the separation of chemotype groups. Second, we employed a cross-validation approach to assess the ability of an LDA classifier to predict chemotype based on the HRM data. Across each of 1000 iterations, an LDA model classifier was created using a randomly selected training set corresponding to ~ 20% of the isolates. The training set was sampled within each chemotype group to represent the overall proportion of chemotypes within the larger dataset. Within each iteration, the LDA classifier was used to predict the chemotypes of the test set which was comprised of the remaining isolates. The error was calculated as the proportion of misclassified chemotypes relative to the total number of predictions, and the accuracy was calculated as the proportion of correctly classified chemotypes. The overall error and accuracy were calculated by averaging the LDA predictions across the 1000 iterations. The code used for the LDA analysis is included in Supplementary File S5. All the plots were generated using the R package ggplot2 (v3.4.4)^81^.

### Sensitivity analysis

The limit of detection (LOD) of the multiplex HRM assay was determined by testing a range of DNA template concentrations from the reference isolates. Fungal DNA was serially diluted in molecular biology grade water to achieve the addition of 20, 2, 0.2, 0.02, or 0.002 ng into the 20 µl reaction volume. The HRM protocol was performed as described above except the extension step was increased to 15 s and number of cycles were increased to 35.

## Electronic supplementary material

Below is the link to the electronic supplementary material.

Supplementary Material 1

Supplementary Material 2

Supplementary Material 3

Supplementary Material 4

Supplementary Material 5

## Data Availability

The experimental data and code used for analyses are available in the main article and Supplementary Information files. More information can be requested from the corresponding authors.
