# Supplementary material for: A Multiplex High-Resolution Melting (HRM) assay to differentiate Fusarium graminearum chemotypes
Source: Sci Rep. 2024 Dec 30;14:31680. doi: 10.1038/s41598-024-81131-5 (PMC11685414; doi:10.1038/s41598-024-81131-5)
Supplement: Supplementary file 1 — Supplementary Material 1 [file 41598_2024_81131_MOESM1_ESM.docx]

**Supplementary Information**

**A Multiplex High-Resolution Melting (HRM) Assay to Differentiate *Fusarium graminearum* Chemotypes**

Lovepreet Singh^1^*, Milton T. Drott^2,3^, Hye-Seon Kim^4^, Robert H. Proctor^4^, Susan P. McCormick^4^, J. Mitch Elmore^2,3^*

^1^Department of Agronomy and Plant Genetics, University of Minnesota, St. Paul, MN 55108, USA

^2^Cereal Disease Laboratory, Agricultural Research Service, US Department of Agriculture, St. Paul, MN 55108, USA

^3^Department of Plant Pathology, University of Minnesota, St. Paul, MN 55108, USA

^4^Mycotoxin Prevention and Applied Microbiology, National Center for Agricultural Utilization Research, Agricultural Research Service, US Department of Agriculture, Peoria, IL, 61604, USA

*Corresponding Authors: Lovepreet Singh (<singhlov@umn.edu>) and J. Mitch Elmore ([mitch.elmore@usda.gov](mailto:mitch.elmore@usda.gov))

**Supplementary Table S1.** *Fusarium* isolates used in this study and chemotype determinations based on the multiplex High-resolution Melting (HRM) assay developed in this study and GC-MS or PCR methods used in previous studies.

| **Isolate ID** | **NRRL ID** | **Species** | **Geographic origin** | **Chemotype** | **Detection method^b^** | | **HRM genotype** | **Reference** |
| --- | --- | --- | --- | --- | --- | --- | --- | --- |
|  |  |  |  |  | **PCR** | **GC-MS** |  |  |
| **PH1^a^** | 31084 | *F. graminearum* | Michigan, USA | 15ADON |  | + | 15ADON | ^1^ |
| A2-04-6 | 37408 | *F. graminearum* | Alberta, Canada | 15ADON |  | + | 15ADON | ^2^ |
| M5-04-2 | 37450 | *F. graminearum* | Manitoba, Canada | 15ADON |  | + | 15ADON | ^2^ |
| S5A-04-2 | 37509 | *F. graminearum* | Saskatchewan, Canada | 15ADON |  | + | 15ADON | ^2^ |
| F-60 | 37605 | *F.vorosii* | Ipolydama ́sd, Hungary | 15ADON |  | + | 15ADON | ^3^ |
| G-11 | 38207 | *F.vorosii* | Hokkaido, Japan | 15ADON |  | + | 15ADON | ^3^ |
| G-12 | 38208 | *F.vorosii* | Hokkaido, Japan | 15ADON |  | + | 15ADON | ^3^ |
| **00-500^a^** | 46416 | *F. graminearum* | Minnesota, USA | 3ADON |  | + | 3ADON | ^1^ |
| A1-04-3 | 37401 | *F. graminearum* | Alberta, Canada | 3ADON |  | + | 3ADON | ^2^ |
| M1-04-2 | 37425 | *F. graminearum* | Manitoba, Canada | 3ADON |  | + | 3ADON | ^2^ |
| S8A-04-2 | 37525 | *F. graminearum* | Saskatchewan, Canada | 3ADON |  | + | 3ADON | ^2^ |
| 11MW12 |  |  | North Dakota, USA | 3ADON |  | + | 3ADON | ^1^ |
| 12SD6-2 | 64391 |  | South Dakota, USA | 3ADON |  | + | 3ADON | ^1^ |
| **02-15^a^** |  | *F. graminearum* | Louisiana, USA | NIV |  | + | NIV | ^4^ |
| 03-126 | 38371 | *F. graminearum* | Louisiana, USA | NIV |  | + | NIV | ^4^ |
| 03-29 | 38373 | *F. graminearum* | Louisiana, USA | NIV |  | + | NIV | ^4^ |
| 03-40 | 38402 | *F. graminearum* | Louisiana, USA | NIV |  | + | NIV | ^4^ |
| 03-83 | 38392 | *F. graminearum* | Louisiana, USA | NIV |  | + | NIV | ^4^ |
| 03-112 | 38382 | *F. graminearum* | Louisiana, USA | NIV |  | + | NIV | ^4^ |
| 03-14 | 38401 | *F. graminearum* | Louisiana, USA | NIV |  | + | NIV | ^4^ |
| 03-104 | 38370 | *F.asiaticum* | Louisiana, USA | NIV |  | + | NIV | ^4^ |
| 03-43 | 38384 | *F.asiaticum* | Louisiana, USA | NIV |  | + | NIV | ^4^ |
| 03-51 | 38387 | *F.asiaticum* | Louisiana, USA | NIV |  | + | NIV | ^4^ |
| 03-47 | 38388 | *F.asiaticum* | Louisiana, USA | NIV |  | + | NIV | ^4^ |
| 03-16 | 38400 | *F.asiaticum* | Louisiana, USA | NIV |  | + | NIV | ^4^ |
| B59 | 28439 | *F. graminearum* | Florida, USA | NIV |  | + | NIV | ^3^ |
| 02-224 | 38380 | *F. gerlachii* | Wisconsin, USA | NIV |  | + | NIV | ^3^ |
| HK-3ND-7-17 | 38405 | *F. gerlachii* | North Dakota, USA | NIV |  | + | NIV | ^3^ |
| 00-551 | 36905 | *F. gerlachii* | Minnesota, USA | NIV |  | + | NIV | ^3^ |
| **06-156^a^** | 66038 | *F. graminearum* | Minnesota, USA | NX-2 |  | + | NX-2 | ^1^ |
| 06-146 | 66030 | *F. graminearum* | Minnesota, USA | NX-2 |  | + | NX-2 | ^1^ |
| 06-132 | 66040 | *F. graminearum* | Minnesota, USA | NX-2 |  | + | NX-2 | ^1^ |
| 00-556 | 66047 | *F. graminearum* | Minnesota, USA | NX-2 |  | + | NX-2 | ^1^ |
| 00-552 | 66049 | *F. graminearum* | Minnesota, USA | NX-2 |  | + | NX-2 | ^1^ |
| ON-06-4 | 43884 | *F. graminearum* | Ontario, Canada | NX-2 |  | + | NX-2 | ^5^ |
| ON-06-190 | 44070 | *F. graminearum* | Ontario, Canada | NX-2 |  | + | NX-2 | ^5^ |
| ON-06-198 | 44078 | *F. graminearum* | Ontario, Canada | NX-2 |  | + | NX-2 | ^5^ |
| S5B-06-4 | 44211 | *F. graminearum* | Saskatchewan, Canada | NX-2 |  | + | NX-2 | ^5^ |
| Q-06-67 | 45156 | *F. graminearum* | Quebec, Canada | NX-2 |  | + | NX-2 | ^5^ |
| Q-07-38 | 47605 | *F. graminearum* | Quebec, Canada | NX-2 |  | + | NX-2 | ^5^ |
| Q-07-92 | 47659 | *F. graminearum* | Quebec, Canada | NX-2 |  | + | NX-2 | ^5^ |
| PEI-07-15 | 53173 | *F. graminearum* | Prince Edward Island, Canada | NX-2 |  | + | NX-2 | ^5^ |
| 04-188 | 66043 | *F. graminearum* | Minnesota, USA | NX-2 |  | + | NX-2 | ^1^ |
| 03-279 | 66045 | *F. graminearum* | North Dakota, USA | NX-2 |  | + | NX-2 | ^1^ |
| 03-348 | 66044 | *F. graminearum* | North Dakota, USA | NX-2 |  | + | NX-2 | ^1^ |
| 04-308 | 66042 | *F. graminearum* | North Dakota, USA | NX-2 |  | + | NX-2 | ^1^ |
| 04-322 | 66041 | *F. graminearum* | North Dakota, USA | NX-2 |  | + | NX-2 | ^1^ |
| 12MN1-3 | 66031 | *F. graminearum* | Minnesota, USA | NX-2 |  | + | NX-2 | ^1^ |
| 00-355 |  |  | South Dakota, USA | 15ADON | + |  | 15ADON | ^6^ |
| 00-597 |  |  | Minnesota, USA | 15ADON | + |  | 15ADON | ^6^ |
| 06-219 | 64385 | *F. graminearum* | North Dakota, USA | 15ADON | + |  | 15ADON | ^6^ |
| 06-225 | 64386 | *F. graminearum* | North Dakota, USA | 15ADON | + |  | 15ADON | ^6^ |
| 06-238 | 64388 | *F. graminearum* | North Dakota, USA | 15ADON | + |  | 15ADON | ^6^ |
| 06-267 |  |  | South Dakota, USA | 15ADON | + |  | 15ADON | ^6^ |
| 06-270 |  |  | South Dakota, USA | 15ADON | + |  | 15ADON | ^6^ |
| 11MW14 | 64389 | *F. graminearum* | South Dakota, USA | 15ADON | + |  | 15ADON | ^6^ |
| 13MN1-6 | 64387 | *F. graminearum* | Minnesota, USA | 15ADON | + |  | 15ADON | ^6^ |
| Q-07-4 | 47571 | *F. graminearum* | Quebec, Canada | 15ADON | + |  | 15ADON | ^6^ |
| M6-07-28 | 52005 | *F. graminearum* | Manitoba, Canada | 15ADON | + |  | 15ADON | ^6^ |
| S1A-07-1 | 52129 | *F. graminearum* | Saskatchewan, Canada | 15ADON | + |  | 15ADON | ^6^ |
| A2-07-91 | 52512 | *F. graminearum* | Alberta, Canada | 15ADON | + |  | 15ADON | ^6^ |
| NB-07-8 | 52840 | *F. graminearum* | New Brunswick, Canada | 15ADON | + |  | 15ADON | ^6^ |
| NB-07-18 | 52850 | *F. graminearum* | New Brunswick, Canada | 15ADON | + |  | 15ADON | ^6^ |
| ON-07-18 | 52955 | *F. graminearum* | Ontario, Canada | 15ADON | + |  | 15ADON | ^6^ |
| 00-837 | 46434 | *F. sp.* | North Dakota, USA | 3ADON | + |  | 3ADON | ^6^ |
| 00-588 | 46426 | *F. graminearum* | Minnesota, USA | 3ADON | + |  | 3ADON | ^6^ |
| 06-193 |  |  | Minnesota, USA | 3ADON | + |  | 3ADON | ^6^ |
| 06-199 |  |  | Minnesota, USA | 3ADON | + |  | 3ADON | ^6^ |
| 06-228 |  |  | North Dakota, USA | 3ADON | + |  | 3ADON | ^6^ |
| 06-239 | 64392 | *F. graminearum* | North Dakota, USA | 3ADON | + |  | 3ADON | ^6^ |
| 06-240 | 64393 | *F. graminearum* | North Dakota, USA | 3ADON | + |  | 3ADON | ^6^ |
| Q-07-21 | 47588 | *F. graminearum* | Quebec, Canada | 3ADON | + |  | 3ADON | ^6^ |
| M7-07-1 | 52008 | *F. graminearum* | Manitoba, Canada | 3ADON | + |  | 3ADON | ^6^ |
| S2A-07-7 | 52195 | *F. graminearum* | Saskatchewan, Canada | 3ADON | + |  | 3ADON | ^6^ |
| A2-07-8 | 52429 | *F. graminearum* | Alberta, Canada | 3ADON | + |  | 3ADON | ^6^ |
| NB-07-15 | 52847 | *F. graminearum* | New Brunswick, Canada | 3ADON | + |  | 3ADON | ^6^ |
| ON-07-109 | 53046 | *F. graminearum* | Ontario, Canada | 3ADON | + |  | 3ADON | ^6^ |
| PEI-07-26 | 53184 | *F. graminearum* | Prince Edward Island, Canada | 3ADON | + |  | 3ADON | ^6^ |
| M11-05-oat4 | 43161 | *F. graminearum* | Manitoba, Canada | NX-2 | + |  | NX-2 | ^6^ |
| B-8 | 45373 | *F. graminearum* | Connecticut, USA | NX-2 | + |  | NX-2 | ^6^ |

^a^Standard reference isolates used for assay development.

^b^ “+” designates the method used by previous studies for determining the chemotype of each isolate.

**Supplementary Figure S1.** Derivative melt plots of the reference isolates (PH-1, 15-ADON; 00-500, 3-ADON; 02-15, NIV; 06-156, NX-2) and the three *F. vorosii* isolates (NRRL 37605, 38207, 38208) with a shifted TRI8_15-ADON peak.


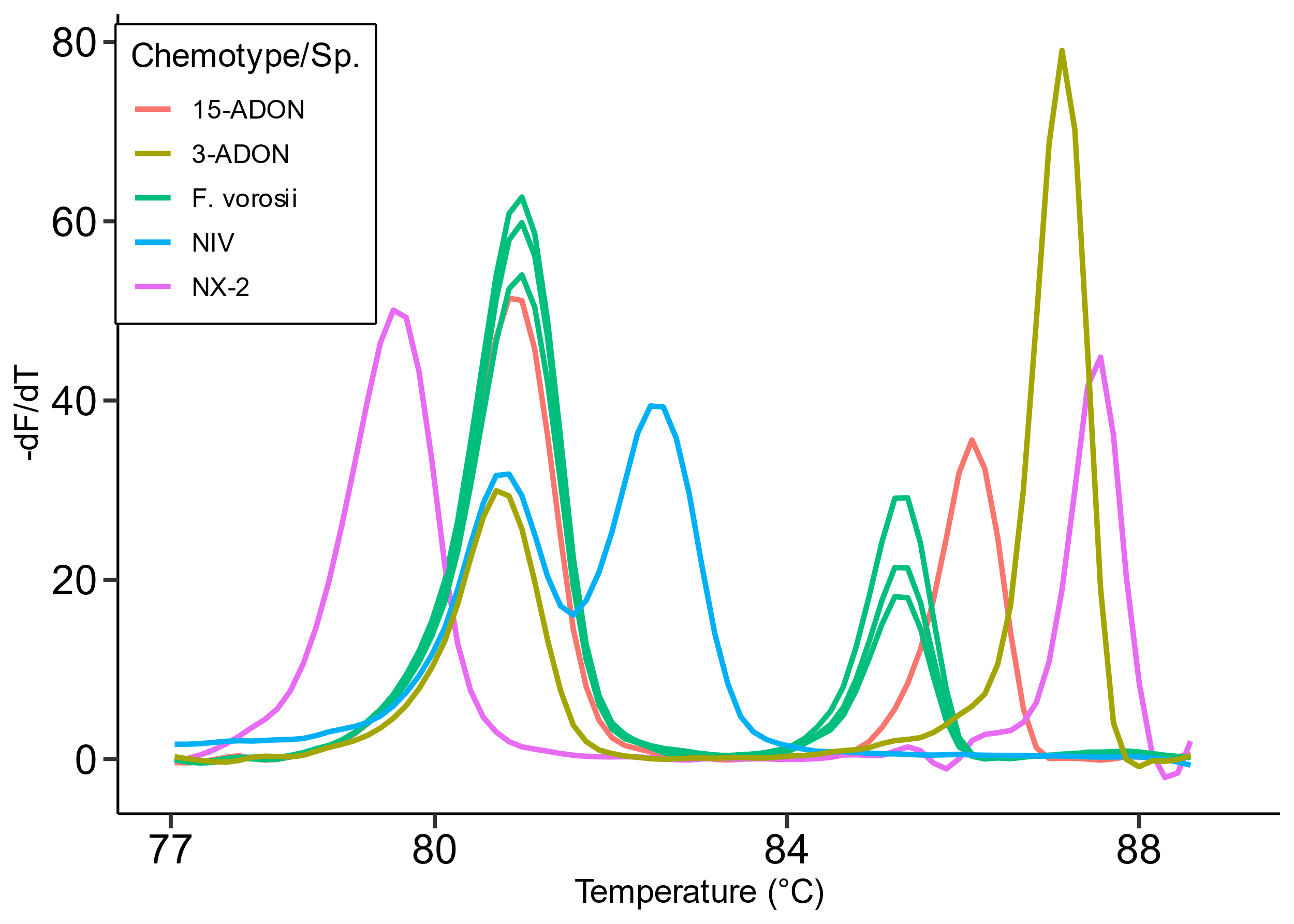


**References**

1. Liang, J. M. *et al.* Temporal dynamics and population genetic structure of *Fusarium graminearum* in the upper Midwestern United States. *Fungal Genet. Biol.* **73**, 83–92 (2014).

2. Ward, T. J. *et al.* An adaptive evolutionary shift in Fusarium head blight pathogen populations is driving the rapid spread of more toxigenic *Fusarium graminearum* in North America. *Fungal Genet. Biol.* **45**, 473–484 (2008).

3. Starkey, D. E. *et al.* Global molecular surveillance reveals novel Fusarium head blight species and trichothecene toxin diversity. *Fungal Genet. Biol.* **44**, 1191–1204 (2007).

4. Gale, L. R. *et al.* Nivalenol-Type Populations of *Fusarium graminearum* and *F. asiaticum* Are Prevalent on Wheat in Southern Louisiana. *Phytopathology* **101**, 124–134 (2011).

5. Kelly, A. C. *et al.* Diversity of Fusarium head blight populations and trichothecene toxin types reveals regional differences in pathogen composition and temporal dynamics. *Fungal Genet. Biol.* **82**, 22–31 (2015).

6. Kelly, A. C. & Ward, T. J. Population genomics of *Fusarium graminearum* reveals signatures of divergent evolution within a major cereal pathogen. *PLOS ONE* **13**, e0194616 (2018).
